# The Relationship between the Presence of an Earlobe Crease and Overactive Bladder: A Cross-Sectional Case-Controlled Study

**DOI:** 10.3390/medicina59030476

**Published:** 2023-02-28

**Authors:** Yasufumi Ueda, Tomohiro Matsuo, Ken Kawada, Hidenori Ito, Kensuke Mitsunari, Kojiro Ohba, Ryoichi Imamura

**Affiliations:** Department of Urology, Nagasaki University Graduate School of Biomedical Sciences, Nagasaki 852-8501, Japan

**Keywords:** overactive bladder, earlobe crease, lower urinary tract symptom

## Abstract

*Background and Objectives*: To examine the relationship between the presence of earlobe crease (EC) and overactive bladder (OAB). *Materials and Methods*: The earlobes of the participants were examined macroscopically. ECs were further divided into four groups (grades 0–3) according to severity. Subjective symptoms were assessed using the OAB symptom score (OABSS), and objective findings were assessed using uroflowmetry. The relationship between these findings and the presence or absence and severity of EC was also examined. A score of ≥2 points on OABSS question 3 (urinary urgency), with a total score of ≥3 points, indicated OAB. *Results*: We analyzed 246 participants, including 120 (48.8%) in the EC group and 126 (51.2%) in the non-EC (N-EC) group. On the OABSS, the EC group scored higher than the N-EC group for all questions and for the total score. The total OABSS of EC grade 3 was the highest of all groups. A total of 115 (95.8%) patients in the EC group (100% in grade 3) and 69 (54.8%) in the N-EC group met the OAB criteria (*p* < 0.001). The voided volume and maximum flow rate of the EC group were significantly lower than those of the N-EC group (both *p* < 0.001). The post-void residual urine volume in the EC group was significantly higher than that in the N-EC group (*p* = 0.029). Multivariate analysis revealed that EC was an independent risk factor for OAB (odds ratio, 8.15; 95% confidence interval, 2.84–24.75; *p* < 0.001). *Conclusions*: The presence of an earlobe crease may be a predictive marker for OAB.

## 1. Introduction

Overactive bladder (OAB) is a symptom-based syndrome characterized by urinary urgency with or without urge incontinence [1]. Some epidemiological reports suggest that the prevalence of OAB ranges between 16 and 19% and increases with age [2,3,4]. In addition, it has been proven that metabolic syndrome and lifestyle-related diseases such as hypertension and diabetes mellitus induce lower urinary tract symptoms (LUTS), including OAB [5]. Furthermore, excessive oxidative stress, which is induced by general comorbidities, causes bladder ischemia due to pelvic artery insufficiency and LUTS such as OAB [6].

Earlobe creases (ECs), also known as “Frank’s sign”, were first reported by Sanders T. Frank as one of the markers for cardiovascular disease [7] and are also associated with metabolic syndrome which causes angiopathic changes in the arterioles [8]. Previous studies have identified the presence of an EC as a risk factor for ischemic heart disease, and an EC is a biomarker for chronic systemic inflammation and excessive oxidative stress [9].

Metabolic syndrome and lifestyle-related diseases that may induce systemic oxidative stress are considered to be related to the presence of OAB and EC. However, no report has clarified the relationship between these conditions, which have some commonalities in their onset.

Hence, this study aimed to primarily examine the relationship between the prevalence of OAB and the presence of ECs. Additionally, we evaluated whether the presence and severity of ECs could affect the prevalence of OAB, using statistical methods.

## 2. Materials and Methods

### 2.1. Patients

**Figure 1:**
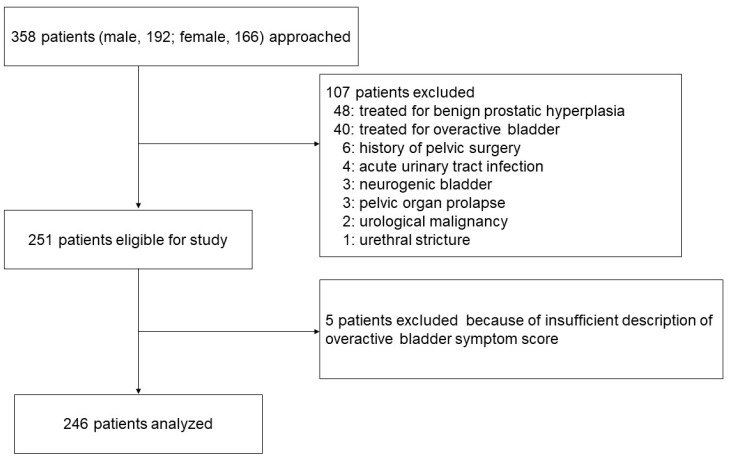
Patient flow diagram.

Our study enrolled new patients who presented to Nagasaki University Hospital with at least one LUTS between January 2022 and October 2022. We included a total of 358 consecutive patients (192 males, 166 females) who met our inclusion criteria. The exclusion criteria included patients who were already being treated for OAB, benign prostatic hyperplasia, acute urinary tract infection, or any condition affecting urinary function, including a history of pelvic surgery, urethral stricture, pelvic organ prolapse, urological malignancy, or neurogenic bladder (Figure 1).

Patients were divided into two groups based on the presence (EC group) or absence (N-EC group) of ECs. This study was approved by the Nagasaki University Hospital Ethical Committee (#20122138) and carried out in accordance with the principles of the Declaration of Helsinki. All participants provided written informed consent.

### 2.2. Evaluation of Vital Signs

Hypertension was defined as a systolic blood pressure ≥140 mmHg and/or a diastolic blood pressure ≥90 mmHg measured using an automated oscillometric upper-arm BP monitoring device (HBP-9020, Omron Co., Kyoto, Japan) by well-trained medical nurses or as receiving therapy for hypertension. Body height (cm) and weight (kg) were recorded and used to calculate the body mass index (BMI) using the standard formula (BMI = weight [kg]/height [m]^2^). Renal dysfunction was defined as an estimated glomerular filtration rate <60 mL/min/1.73 m^2^. Determination of BMI values and the presence of renal dysfunction were performed by two investigators (T.M. and H.I.).

### 2.3. Evaluation of Earlobe Crease

**Figure 2:**
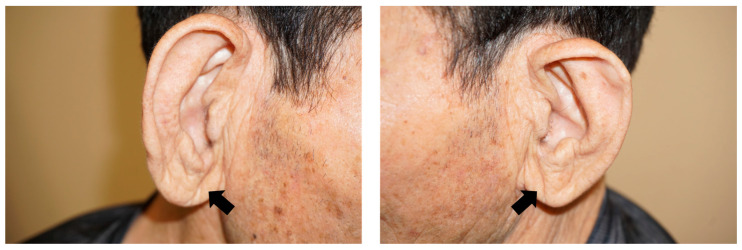
Representative pictures of grade 3 earlobe crease (arrows).

The presence or absence of EC was evaluated in accordance with previous studies [10,11]. In brief, bilateral earlobe photographs were taken by one researcher with the patient in a seated position. Each digital photo was assessed independently by two researchers (H.I. and K.M.) who were blinded to each other’s assessments and to the clinical data to determine the presence of EC. Positive EC was considered when subjects had a crease or wrinkle extending 45° diagonally from the tragus toward the outer border of the earlobe. Discrepancies were resolved by reaching a consensus through the participation of a third researcher. In addition, based on a previous study, EC was classified into four categories [12]. Briefly, each EC was graded according to its length (0, no EC; 1, <50% diagonal width of the earlobe; 2, 51%–90% diagonal width of the earlobe; 3, >90% diagonal width of the earlobe) [12]. (Figure 2). The EC length was measured using the ImageJ software (National Institute of Health [NIH], Bethesda, MD, USA), based on previously taken photographs.

### 2.4. Evaluation of Lower Urinary Tract Symptoms

Based on the OAB symptom score (OABSS), we defined participants with a urinary urgency (Question [Q]3) score ≥2 and those with a total score ≥3 as having OAB [13]. In addition, we evaluated objective urinary symptoms using uroflowmetry (AQUARIUS^®^CTS; EDAP TMS Japan, Tokyo, Japan). Furthermore, the post-void residual urine volume was measured using ultrasound examination (Hi VISION Avius; Hitachi Medical Corporation, Tokyo, Japan). 

### 2.5. Statistical Analyses

All data are presented as mean ± standard deviation. The Student’s *t*-test and the Mann–Whitney U test were used to evaluate changes in subjective and objective symptoms, as required. Moreover, the χ^2^-test was used for categorical comparisons. Crude and adjusted effects were estimated using logistic regression analysis and described as odds ratios (ORs) with 95% confidence intervals (CIs), along with *p*-values. All tests were two-sided, and statistical significance was set at *p* < 0.05. All statistical analyses were performed using the JMP 15 software (SAS Institute, Cary, NC, USA).

The study sample size was determined using the G*Power version 3.1 software based on previous reports, with a probability of 0.05 (two-sided), a power of 80%, and an effect size of 0.5. The estimated ideal number of participants for the study was at least 244.

### 2.6. Propensity Score Matching

Based on their characteristics, patients with OAB were matched (1:1 ratio) with non-OAB patients according to their propensity score through nearest-neighbor matching. A caliper width of 0.2 SDs was used.

## 3. Results

### 3.1. Patient Characteristics

We analyzed 246 individuals (125 men, 50.8%), including 184 (74.8%) with OAB. Age and the proportion of the comorbidities hypertension, diabetes mellitus, hyperlipidemia, and chronic renal dysfunction were significantly higher in the OAB group than in the non-OAB group (Table 1). In addition, all four items and the total OABSS were higher in the OAB group compared to the non-OAB group. In contrast, the voided volume and maximum flow rate in the OAB group were lower than those in the non-OAB group (Table 1).

Of the 246 participants, 120 (48.8%) were included in the EC group and 126 (51.2%) in the non-EC (N-EC) group (Table 2). The mean patient age was 68.7 ± 13.1 years, and there were significant differences between the EC and N-EC groups (*p* < 0.001). Compared to the N-EC group, the EC group had a higher proportion of males (*p* < 0.001) and patients with hypertension (*p* < 0.001), diabetes mellitus (*p* < 0.001), hyperlipidemia (*p* < 0.001), and chronic renal dysfunction (*p* < 0.001) (Table 2). 

### 3.2. Differences in Urological Parameters between EC and N-EC Groups

Table 3 shows the differences in subjective urological symptoms and objective findings between the two groups. Regarding the OABSS, the EC group scored higher than the N-EC group on all four questions and on the total score. Overall, 115 (95.8%) patients in the EC group and 69 (54.8%) in the N-EC group met the OAB criteria, with a predominance in the EC group (*p* < 0.001).

In the objective findings, the voided volume (EC group, 149.9 ± 47.9 mL; N-EC group, 200.1 ± 73.8 mL; *p* < 0.001) and maximum flow rate (EC group, 14.3 ± 14.0 mL/s; N-EC group, 21.2 ± 9.2 mL/s; *p* < 0.001) of the EC group were significantly lower than those of the N-EC group. Additionally, the post-void residual urine volume (EC group, 27.9 ± 23.5 mL; N-EC group, 22.1 ± 17.1 mL; *p* = 0.029) of the EC group was significantly higher than that of the N-EC group.

### 3.3. Differences in Patient Characteristics, Related to the Severity of EC

Table 4 shows the differences in patient backgrounds among the four groups classified by EC severity. Regardless of the severity of EC, the proportion of men with EC (EC grades 1, 2, and 3) was higher than that of men without EC (EC grade 0) (all *p* < 0.001). In addition, compared with EC grade 0, there were higher proportions of patients with hypertension, diabetes, hyperlipidemia, and chronic renal dysfunction for all EC grades (Table 4). However, there were no significant differences among the three groups with EC regarding age and proportion of participants with hypertension, diabetes mellitus, hyperlipidemia, and chronic renal dysfunction (Table 4).

### 3.4. Differences in Subjective Symptoms and Objective Findings among the EC Groups

Table 5 shows the relationship between EC grade and urological parameters. In terms of subjective symptoms using the OABSS, the symptoms of all EC groups were more severe than those of the N-EC groups (EC grade 0) in all OABSS items and total score, regardless of EC severity (Table 5). In Q3 (urgency) and Q4 (urgency incontinence), the scores of the EC grade 2 and 3 groups were also higher than those of the EC grade 1 group. Furthermore, the total OABSS of EC grade 3 was the highest of all groups. In addition, patients in the EC groups met the OAB definition more than those in the N-EC group (Table 5). Particularly, the individuals who met the criteria for OAB included 100% of the EC grade 3 group.

The voided volume was lower in all three EC groups compared to the N-EC group (all *p* < 0.001). Regarding the maximum flow rate, EC grades 1, 2, and 3 were lower values than grade 0 (EC grade 0 vs. EC grade 1, *p* = 0.009; EC grade 0 vs. EC grade 2, *p* < 0.001; EC grade 0 vs. EC grade 3, *p* < 0.001), and EC grade 1 had significantly more residual urine volume than EC grade 0 (*p* = 0.001).

### 3.5. Overactive Bladder and Urinary Symptoms-Related Factors

Based on these results, we conducted univariate and multivariate analyses to clarify the independent risk factors of age, sex, body mass index, general comorbidity, and EC on OAB. We found that EC, sex, and hypertension were independent risk factors for the prevalence of OAB (Table 6). In addition, we further investigated whether EC could be a predictor of OAB by itself using propensity score matching scores (Table 7 and Table 8).

Data from 108 patients (54 in each group) were included in this analysis. Standardized mean differences for all characteristics were less than 0.1, indicating that baseline differences between groups were negligible. Patients with EC were significantly higher in the OAB patient group (*p* = 0.004; 95% confidence interval, 1.66–14.40; odds ratio, 4.90) (Table 8).

## 4. Discussion

This clinical study showed that the presence of EC is related to age and lifestyle-related diseases such as hypertension. Additionally, individuals with EC were more likely to have OAB than those without, and their OAB symptoms were more severe both subjectively and objectively. However, in the present study, the severity of EC did not significantly affect OAB symptoms.

EC was more prevalent in males than females in our study. In addition, more patients in the EC group were older and had a higher BMI and various systemic comorbidities compared to patients in the N-EC group. Similar to our observations, Kang et al. [8], whose study included participants aged 20–79 years, found that EC was more common in males and that the proportion of patients with EC increased with age. Although few studies have reported no difference in the occurrence of EC between sexes [14,15], which is debatable, there seems to be a certain consensus on the occurrence of EC and age [10,14,15]. A detailed examination of the present study did not always reveal a correlation between the four EC grades and age. However, in a previous study that divided EC grade into two groups, the mean age of the grade 2/3 group (70.60 years) was significantly higher than that of the EC grade 0/1 group (62.43 years) [16]. Furthermore, in the present study, as shown in Table 4, the mean age of patients with EC grade 2/3 was higher than that of patients with EC grade 0/1, and we believe that the trend between EC and age can be moderately recognized. Therefore, there may also be a relationship between aging and EC severity.

An association has been shown between EC and the prevalence of systemic comorbidities [8,17] that could also cause OAB. Similar to previous studies, we found an association between the presence of EC and general comorbidities. However, an association between EC severity and the incidence of comorbidities was not observed. Furthermore, we did not examine the treatment of comorbidities or the relationship between efficacy and EC in detail in this study. Previous studies have not investigated the relationship between EC severity and comorbidities in detail either; thus further studies are required.

Some researchers consider EC to be a potential dermatological marker of systemic atherosclerosis; however, the detailed mechanisms of its development are not known [18]. EC occurs via vascular endothelial dysfunction, which is also associated with the development of coronary vascular disease and other conditions [14,18]. Furthermore, EC associated with atherosclerosis may also be induced by skin aging, collagen degeneration, and telomere shortening [19,20,21,22]. The EC lesion induced by these consequences ultimately alters the shape of the ear, through the progression of ischemia-induced fibrosis [23].

Both basic and clinical studies have revealed a similar mechanism for the development of OAB, which is caused by ischemia in pelvic organs, especially the bladder; specifically, ischemia and the resulting excessive oxidative stress cause detrusor hyperactivity, which eventually induces fibrosis and changes the shape of the bladder [6,24]. Interestingly, although the pathogenesis of EC and OAB are not fully understood, especially given their different sites of origin, the two conditions may have a common underlying pathology associated with ischemia. This is our hypothesis, and it requires further and detailed investigation. In the present study, more patients in the EC group met the diagnostic criteria for OAB than those in the N-EC group. Additionally, all OABSS questionnaires and total scores of the EC groups were worse than those of the N-EC group. The number of patients with OAB increased with the severity of EC, and all patients in the EC grade 3 group met the diagnostic criteria for OAB. Furthermore, OAB symptoms were worse in EC grades 2 and 3 than in EC grade 1, and the total score was significantly higher in grade 3 than in grades 1 and 0. In addition, EC grade 3 was higher than EC grade 2 in terms of the total OABSS score, although the difference was not statistically significant. Therefore, we believe that there is a relationship between the presence of EC, its severity, and the subjective symptoms of OAB. However, no previous study has examined the relationship between EC grade and disease severity in detail, and, to the best of our knowledge, this is the first attempt to clarify this problem. In a previous study by Thilo et al., a higher proportion of patients with hypertension, dyslipidemia, renal dysfunction, coronary vascular disease, heart failure, and serum C-reactive protein levels were found in the EC grade 2/3 group than in the EC grade 0/1 group [16]. Their results indicate that both the presence of EC and the magnitude of its grade are associated with the risk of developing systemic diseases. Moreover, Oda et al. divided ECs into five categories according to the severity of endothelial dysfunction [14] and showed that endothelial functions, such as flow-mediated vasodilation and nitroglycerine-induced vasodilation, decreased according to EC severity. These results support our assumption that the severity of EC is associated with an increase in the number of patients with OAB and the worsening of subjective symptoms.

In contrast, the voided volume and maximum flow rate in the EC group were lower than those in the N-EC group. However, neither seemed to be related to EC severity. Furthermore, only the EC grade 1 group had significantly more post-void residual urine than the EC grade 0 group. Although future detailed studies are required, the results of this study suggest that voiding efficacy and post-void residual urine may worsen in individuals with EC. In particular, the use of anticholinergic agents in elderly patients with OAB may cause dysuria as an adverse event and increase residual urine [25]. Furthermore, it has been noted that the presence of EC could also be associated with cognitive dysfunction, which may occur as an adverse event of anticholinergic drugs [26,27]. Therefore, the presence of EC in patients with OAB may indicate potential dysuria and cognitive dysfunction, and might be a surrogate marker for the development of safer treatment strategies for OAB.

The present study had several limitations that should be acknowledged. First, its sample size was relatively small. As there have been no previous studies on the prevalence of EC and OAB, the number of participants recruited was set with reference to a previous study [12,19] on EC and various systemic comorbidities. Additionally, we used a convenience sampling method, given that participants were recruited from a specific population (new patients who presented to Nagasaki University Hospital). Convenience sampling is a non-probability sampling technique that involves selecting participants based on their availability and willingness to participate. However, this type of sampling can introduce bias as it may not be representative of the broader population. Thus, sampling bias may have been introduced in this study, and our findings may not be generalizable to the broader population. It is therefore essential to design a larger and more thorough study that enables a detailed analysis of the relationship between EC grade and OAB severity, which could not be clarified by this study. 

There are further limitations associated with this research that similar studies should address in the future. For instance, only patients with LUTS were recruited in this study, and control individuals without LUTS were not included. In addition, the severity and treatment of systemic comorbidities may have influenced the observations of this study. However, details of oxidative stress and vascular endothelial function have not yet been investigated. Moreover, as we mainly investigated the relationship with OAB, we did not examine voiding symptoms in detail. Therefore, a survey using a detailed questionnaire including the International Prostate Symptom Score is necessary. Furthermore, in the objective findings, a detailed pressure-flow study must be conducted. Despite these issues and the fact that strong correlations between the degree of EC and OAB severity were not always present in this study, we determined that the presence of EC was not only a univariate and multivariate factor but also showed that sex and hypertension were independent risk factors for OAB. Furthermore, the presence of EC was identified as a significant risk factor for OAB using propensity score matching, which excluded the effects of gender and systemic comorbidities.

## 5. Conclusions

To our knowledge, this study is the first to clarify the relationship between EC and OAB. In this study, the degree of EC and the severity of OAB did not always match perfectly. However, in the present report, it is the strong point that we found EC is an important risk factor for the development of OAB by using propensity score matching as well as multivariate analysis. Furthermore, we have shown that the presence of ECs affects the decrease in the urinary stream and voided volume in objective findings using uroflowmetry. However, the relationship between EC grade and the severity of objective findings as well as that of subjective symptoms was unclear. Based on the above results, in the future, it will be necessary to conduct a study that includes a detailed evaluation of the differences in bladder function between patients with and without EC.

## Figures and Tables

**Table 1:** General patient characteristics and differences between the two groups with or without OAB.

	Entire	OAB Group	Non-OAB Group	*p*-Value
No. of participants (male, %)	246 (125, 50.8)	184 (105, 57.1)	62 (20, 32.3)	0.001
Age (years)	68.7 ± 13.1	70.7 ± 11.7	62.9 ± 15.3	<0.001
Body mass index (kg/m^2^)	23.5 ± 4.1	22.7 ± 3.8	23.7 ± 4.2	0.067
Earlobe crease	120 (48.8)	115 (62.5)	5 (8.1)	<0.001
Hypertension	126 (51.2)	106 (57.6)	20 (32.3)	<0.001
Diabetes mellitus	59 (24.0)	53 (28.8)	6 (9.7)	0.002
Hyperlipidemia	53 (21.5)	46 (25.0)	7 (11.3)	0.031
Chronic renal dysfunction	98 (39.8)	85 (46.2)	13 (21.0)	<0.001
OABSS				
Q1 Daytime frequency	0.8 ± 0.7	1.0 ± 0.8	0.3 ± 0.5	<0.001
Q2 Nocturia	2.0 ± 1.0	2.3 ± 0.7	1.1 ± 1.0	<0.001
Q3 Urgency	2.5 ± 1.5	3.2 ± 0.9	0.5 ± 0.7	<0.001
Q4 Urgency incontinence	1.5 ± 1.5	1.9 ± 1.4	0.4 ± 0.9	<0.001
Total score	6.9 ± 3.8	8.5 ± 2.5	2.3 ± 3.2	<0.001
Urodynamic study				
Voided volume (mL)	175.6 ± 67.3	156.9 ± 51.9	231.1 ± 76.9	<0.001
Maximum flow rate (mL/s)	17.9 ± 12.3	16.4 ± 13.1	22.3 ± 8.2	<0.001
PVR (mL)	24.9 ± 20.6	26.0 ± 21.6	21.9 ± 17.1	0.226

Data are presented as mean ± standard deviation and n (%). OAB, overactive bladder; OABSS, overactive bladder symptom score; PVR, post-void residual urine.

**Table 2:** General patient characteristics and differences between the two groups with and without EC.

	Entire	EC Group	N-EC Group	*p*-Value
No. of participants (male, %)	246 (125, 50.8)	120 (91, 75.8)	126 (34, 27.0)	<0.001
Age (years)	68.7 ± 13.1	73.1 ± 8.5	64.6 ± 15.2	<0.001
Body mass index (kg/m^2^)	23.5 ± 4.1	23.8 ± 3.8	23.1 ± 4.3	0.041
Comorbidity				
Hypertension	126 (51.2)	84 (70.0)	42 (33.3)	<0.001
Diabetes mellitus	59 (24.0)	43 (35.8)	16 (12.7)	<0.001
Hyperlipidemia	53 (21.5)	38 (31.7)	15 (11.9)	<0.001
Chronic renal dysfunction	98 (39.8)	72 (60.0)	26 (20.6)	<0.001

Data are presented as mean ± standard deviation and n (%). EC, earlobe crease; N-EC, non-earlobe crease.

**Table 3:** Differences in urological parameters.

	Entire	EC Group	N-EC Group	*p*-Value
OABSS				
Q1 Daytime frequency	0.8 ± 0.7	1.1 ± 0.7	0.6 ± 0.6	<0.001
Q2 Nocturia	2.0 ± 1.0	2.5 ± 0.6	1.5 ± 1.0	<0.001
Q3 Urgency	2.5 ± 1.5	3.2 ± 1.1	2.0 ± 1.6	<0.001
Q4 Urgency incontinence	1.5 ± 1.5	2.1 ± 1.5	1.0 ± 1.3	<0.001
Total score	6.9 ± 3.8	8.8 ± 2.8	5.1 ± 3.8	<0.001
No. of patients with OAB	184 (74.8)	115 (95.8)	69 (54.8)	<0.001
Urodynamic study				
Voided volume (mL)	175.6 ± 67.3	149.9 ± 47.9	200.1 ± 73.8	<0.001
Maximum flow rate (mL/s)	17.9 ± 12.3	14.3 ± 14.0	21.2 ± 9.2	<0.001
PVR (mL)	24.9 ± 20.6	27.9 ± 23.5	22.1 ± 17.1	0.029

Data are presented as mean ± standard deviation and n (%). EC, earlobe crease; N-EC, non-earlobe crease; OAB, overactive bladder; OABSS, overactive bladder symptom score; PVR, post-void residual urine volume.

**Table 4:** Relationship between the grade of EC and patient backgrounds.

	EC Grade 0 (N-EC)	EC Grade 1	EC Grade 2	EC Grade 3	*p*-Value
No. of participants (male, %)	126 (34, 27.0)	32 (26, 81.3) **	53 (39, 73.6) **	35 (26, 74.3) **	<0.001
Age (years)	64.6 ± 15.2	70.2 ± 12.0 *	75.0 ± 6.8 **	72.8 ± 6.2 **	<0.001
Body mass index (kg/m^2^)	23.1 ± 4.3	24.6 ± 4.2	23.4 ± 4.1	23.8 ± 3.1	0.298
Comorbidity					
Hypertension	42 (33.3)	21 (65.6) **	43 (81.1) **	20 (57.1) **	<0.001
Diabetes mellitus	16 (12.7)	13 (40.6) **	18 (34.0) **	12 (34.3) **	<0.001
Hyperlipidemia	15 (11.9)	13 (40.6) **	15 (28.3) **	10 (28.6) *	<0.001
Chronic renal dysfunction	26 (20.6)	18 (56.3) **	32 (60.4) **	22 (62.9) **	0.002

Data are presented as mean ± standard deviation and n (%). EC, earlobe crease; N-EC, non-earlobe crease; *, <0.05 vs. Grade 0; **, <0.01 vs. Grade 0.

**Table 5:** Relationship between the grade of EC and urological symptoms.

	EC Grade 0 (N-EC)	EC Grade 1	EC Grade 2	EC Grade 3	*p*-Value
OABSS					
Q1 Daytime frequency	0.6 ± 0.6	1.0 ± 0.7 **	1.1 ± 0.8 **	1.1 ± 0.7 **	<0.001
Q2 Nocturia	1.5 ± 1.0	2.6 ± 0.6 **	2.5 ± 0.5 **	2.5 ± 0.7 **	<0.001
Q3 Urgency	2.0 ± 1.6	2.7 ± 1.1 **	3.3 ± 1.0 **^,†^	3.5 ± 0.9 **^,††^	<0.001
Q4 Urgency incontinence	1.0 ± 1.3	1.4 ± 1.4 *	2.2 ± 1.3 **^,†^	2.5 ± 1.7 **^,††^	<0.001
Total score	5.1 ± 3.8	7.7 ± 2.6 **	9.0 ± 2.6 **	9.6 ± 2.9 **^,†^	<0.001
No. of patients with OAB	69 (54.8)	29 (90.6) **	51 (96.2) **	35 (100.0) **	<0.001
Urodynamic study					
Voided volume (mL)	200.1 ± 73.8	141.6 ± 42.3 **	142.7 ± 45.5 **	168.5 ± 52.3 **	<0.001
Maximum flow rate (mL/s)	21.2 ± 9.2	15.1 ± 7.1 **	11.9 ± 5.5 **	12.4 ± 6.0 **	<0.001
PVR (mL)	22.1 ± 17.1	35.3 ± 36.7 **	26.6 ± 16.2	23.0 ± 14.7 ^†^	0.011

Data are presented as mean ± standard deviation and n (%). EC, earlobe crease; N-EC, non-earlobe crease; OAB, overactive bladder; OABSS, overactive bladder symptom score; PVR, post-void residual urine volume; *, <0.05 vs. Grade 0; **, <0.01 vs. Grade 0; ^†^, <0.05 vs. Grade 1; ^††^, <0.01 vs. Grade 1.

**Table 6:** OAB and its related factors.

	Univariate Analysis			Multivariate Analysis		
	OR	95% CI	*p*-Value	OR	95% CI	*p*-Value
Gender (male)	2.79	1.54–5.20	<0.001	1.14	1.06–1.23	0.046
Age	1.04	1.02–1.07	0.001	0.98	0.96–1.01	0.251
Body mass index	1.07	0.99–1.15	0.090	-	-	-
Hypertension	2.86	1.57–5.32	<0.001	1.21	1.06–1.34	0.033
Diabetes mellitus	3.78	1.64–10.3	0.004	0.51	0.15–1.47	0.218
Hyperlipidemia	2.62	1.18–6.66	0.017	0.90	0.75–1.18	0.423
Chronic renal dysfunction	3.24	1.69–6.59	<0.001	1.08	0.42–2.75	0.872
EC	10.56	4.32–31.79	<0.001	8.15	2.84–24.75	<0.001

OR, odds ratio; CI, confidence interval; EC, earlobe crease.

**Table 7:** Baseline characteristic data of OAB after propensity score matching.

	Non-OAB N = 54	Earlobe OAB N = 54	*p*-Value	Standardized Mean Differences
Gender (male, %)	19 (35.2)	22 (40.7)	0.692	0.097
Age	65.9 ± 13.3	64.6 ± 14.4	0.618	0.096
Body mass index	22.8 ± 3.9	22.8 ± 4.6	0.964	0.009
Hypertension	20 (37.0)	17 (31.5)	0.685	0.997
Diabetes mellitus	6 (11.1)	7 (13.0)	1.000	0.057
Hyperlipidemia	7 (13.0)	8 (14.8)	0.795	0.074
Chronic renal dysfunction	13 (24.1)	11 (20.4)	0.817	0.089
OAB, overactive bladder				

**Table 8:** Differences in OAB between the presence and absence of EC based on propensity score matching methods.

	Non-OAB N = 54	OAB N = 54	*p*-Value
Earlobe crease (%)	5 (9.3)	19 (35.2)	0.004
OAB, overactive bladder			

## Data Availability

The data presented in this study are available upon request from the corresponding author. The data are not publicly available due to privacy/ethical restrictions.
